# Society Meetings

**Published:** 1892-12

**Authors:** 


The American Orthopedic Association, at its recent meeting, held in the City of New York, September 20, 21, and 22, 1892, elected the following officers to serve for the ensuing year: President, Dr. A. J. Steele, St. Louis; Vice-Presidents, Dr. Samuel Ketch, New York; Dr. Arthur J. Gillette, St. Paul; Treasurer, Dr. A. B. Judson, New York; Secretary, Dr. John Ridlon, 34 Washington street, Chicago. The next annual meeting will be held in St. Louis, the third week in September, 1893.

It is a noteworthy fact (*Medical Review*) that not a single case of small-pox occurred during the year 1890 in the British army. If this be not evidence of the protection afforded by re-vaccination against a malady once so common—and still disastrously fatal in armies where this precaution is not vigorously enforced—then logic and reason are mere accomplishments.—*Ontario Medical Journal.*

